# Automated segmentation of the sacro-iliac joints, posterior spinal joints and discovertebral units on low-dose computed tomography for Na[^18^F]F PET lesion detection in spondyloarthritis patients

**DOI:** 10.1186/s40658-025-00734-7

**Published:** 2025-03-10

**Authors:** Wouter R. P. van der Heijden, Floris H. P. van Velden, Robert Hemke, Tom C. Doorschodt, Ronald Boellaard, Conny J. van der Laken, Gerben J. C. Zwezerijnen

**Affiliations:** 1 Department of Clinical Immunology and Rheumatology, Amsterdam UMC, Meibergdreef 9, Amsterdam, The Netherlands; 2 Department of Radiology and Nuclear Medicine, Amsterdam UMC, De Boelelaan 1117, Amsterdam, The Netherlands; 3 Department of Radiology, Leiden University Medical Center, Albinusdreef 2, Leiden, The Netherlands; 4 Department of Radiology and Nuclear Medicine, OLVG, Oosterparkstraat 9, Amsterdam, The Netherlands

**Keywords:** PET quantification, Automated lesion detection, Artificial intelligence-based segmentation, Rheumatic disease, Spinal bone formation

## Abstract

**Purpose:**

Spondyloarthritis (SpA) is a chronic inflammatory rheumatic disease which involves the axial skeleton. Quantitative sodium fluoride-18 (Na[^18^F]F) PET/CT is a new imaging approach promising for accurate diagnosis and treatment monitoring by assessment of molecular bone pathology in SpA. Detection of Na[^18^F]F PET positive lesions is time-consuming and subjective, and can be replaced by automatic methods. This study aims to develop and validate an algorithm for automated segmentation of the posterior spinal joints, sacro-iliac joints (SIJs) and discovertebral units (DVUs) on low-dose computed tomography (LDCT), and to employ these segmentations for threshold-based lesion detection.

**Methods:**

Two segmentation methods were developed using Na[^18^F]F PET/LDCT images from SpA patients. The first method employed morphological operations to delineate the joints and DVUs, while the second used a multi-atlas-based approach. The performance and reproducibility of these methods were assessed on ten manually segmented LDCTs using average Hausdorff distance (HD) and dice similarity coefficient (DSC) for DVUs and SIJs, and mean error distance for the posterior joints. Various quantitative PET metrics and background corrections were compared to determine optimal lesion detection performance relative to visual assessment.

**Results:**

The morphological method achieved significantly better DSC (0.82 (0.73–0.88) vs. 0.74 (0.68–0.79); *p* < 0.001) for all DVUs combined compared to the atlas-based method. The atlas-based method outperformed the morphological method for the posterior joints with a median error distance of 4.00 mm (4.00–5.66) vs. 5.66 mm (4.00–8.00) (*p* < 0.001). For lesion detection, the atlas-based segmentations were more successful than the morphological method, with the most accurate metric being the maximum standardized uptake value (SUVmax) of the lesional Na[^18^F]F uptake, corrected for the median SUV (SUVmedian) of the spine, with an area under the curve of 0.90.

**Conclusion:**

We present the first methods for detailed automatic segmentation of the posterior spinal joints, DVUs and SIJs on LDCT. The atlas-based method is the most appropriate, reaching high segmentation performance and lesion detection accuracy. More research on the PET-based lesion segmentation is required, to develop a pipeline for fully automated lesional Na[^18^F]F uptake quantification.

**Supplementary Information:**

The online version contains supplementary material available at 10.1186/s40658-025-00734-7.

## Introduction

Spondyloarthritis (SpA) is a chronic inflammatory rheumatic disease that primarily affects the axial skeleton and peripheral entheses, leading to significant morbidity and impaired quality of life [1]. However, diagnosis of SpA is often prolonged due to its gradual symptom development and need for detailed longitudinal evaluations, typically taking years to establish [2, 3]. Early diagnosis of SpA is crucial as it enables an early initiation of effective treatment, ultimately improving patient outcomes [4]. In addition, early efficacy assessment of treatment is needed to prevent prolongation of ineffective treatment with potential side effects and increased risk of progression of structural damage [5]. Sodium fluoride-18 (Na[^18^F]F) PET/CT can effectively detect molecular bone formation activity that matches typical disease locations known for axial SpA, anatomically corresponding to e.g. spinal enthesopathy, syndesmophytes and sacroiliitis [6–9].

Current disease identification and evaluation methodologies using PET/CT depend on qualitative visual assessments and visually guided quantification of identified lesions. Quantification of the lesional PET tracer uptake is superior for assessment and monitoring of rheumatic disease activity than visual interpretation [9, 10]. Visual PET analysis is time-consuming, inherently subjective, and therefore prone to inter- and intra-observer variability [11]. Consequently, there is a growing interest in developing automatic methods that can enhance efficiency and consistency in the quantitative analysis of spinal PET images [12–15]. Extracting various quantitative metrics from PET images, such as standardized uptake value (SUV), and texture or histogram features, provides valuable insights into disease characteristics, heterogeneity and treatment response, enabling clinicians to make more informed decisions. Since the clinical interpretation of Na[^18^F]F PET relies on accurate anatomical localization, a two-step approach is proposed, employing segmentations of the relevant structures using the low-dose CT (LDCT). This approach allows for the analysis of tracer uptake in these anatomical structures, providing the nuclear medicine physicist with a structured report of potential areas of pathological uptake. Fully automated lesion detection has the potential to significantly improve diagnostic accuracy by reducing observer variability and ensuring consistent quantification across patients. Moreover, it can streamline clinical workflows, enabling faster and more reproducible assessments that directly inform patient management and treatment decisions. In SpA, disease activity affects the spinal joints and entheses, often leading to pathologic osteogenesis at these sites [7, 8, 16]. The pathology typically manifests with sacroiliitis, leading to considerable morbidity attributable to pain and reduced mobility [17]. Given this pattern of pathology, the discovertebral unit (DVU), sacro-iliac joints (SIJs) and the posterior joints, specifically the facet joints (FJs), costovertebral joints (CVJs) and the costotransverse joints (CTJs) are the most relevant volumes of interest (VOIs) for PET quantification [18, 19].

While numerous algorithms for automatic segmentation of the axial skeleton on CT have been described [20–23], automatic CT-based segmentation of the posterior vertebral joints, SIJs and DVUs remains a relatively new research area. Piri et al*.* developed a method for segmenting lumbar FJs and intervertebral disks (IVDs) on CT scans of patients with lower back pain [24]. Their approach employed a pre-existent segmentation network RECOMIA for initial segmentation of the vertebrae, followed by morphological dilation and erosion operations to segment the lumbar IVDs and FJs [21]. Several groups have performed automated segmentation or object detection of the SIJs on CT [25–28]. These methods used machine learning to detect structural lesions indicative of SpA. To our knowledge, no automatic segmentation methods for DVUs, CVJs, and CTJs on LDCT have been described currently.

In this paper, we present and compare two automated methods for detailed segmentation of the axial skeleton on LDCT scans. Our primary objective was to develop algorithms that accurately and robustly segment key anatomical components for SpA activity on LDCT, including DVUs, FJs, CVJs, CTJs, and SIJs, facilitating automated Na[^18^F]F PET lesion detection.

## Methods

### Data acquisition

The dataset used to develop the segmentation algorithm consisted of 45 baseline Na[^18^F]F PET/CT scans of pre-treatment SpA patients. The lesion detection performance was validated on a different set consisting of 18 SpA patients who underwent Na[^18^F]F PET/CT at baseline, 12 weeks, and 48 weeks after treatment start. Patients were included if they fulfilled the modified New York criteria and had a high disease activity with a BASDAI score of 4 or higher, or if they had mixed axial and peripheral disease activity including at least two peripheral enthesitis sites [29, 30]. More information about the segmentation dataset has been described in previous publications [7–9]. Whole-body PET images were acquired 45 min after the administration of 101 ± 4 MBq Na[^18^F]F using either Vereos or Ingenuity TF (Philips Healthcare, Best, The Netherlands), or Biograph mCT Flow (Siemens Healthineers, Erlangen, Germany) PET/CT-scanners. The PET scan was preceded by an LDCT scan (30 mAs, 120 kVp).

### Image processing

The initial segmentation of the LDCT was performed using the TotalSegmentator v2 network, which is based on the nn-UNet architecture, which is particularly suitable for biomedical image segmentation [22, 31]. TotalSegmentator had been trained and validated to segment 117 structures on diagnostic CT and LDCT scans. The structures used for further segmentation included individual vertebrae, costae, sacrum, spinal cord and pelvis. After initial segmentation, the PET and segmentation mask were resampled to a resolution of 4 × 4 × 4 mm^3^ using ACCURATE, an in-house developed software tool for PET/CT analysis [32].

### Segmentation

This study encompassed the segmentation of various structures, comprising 24 DVUs, 48 FJs, 24 CVJs, 20 CTJs and 2 SIJs, for which two methods were developed. The first method applied morphological erosion and dilation operations, whereas the second method employed a multi-atlas-based approach. Since the principal application for the joint segmentation algorithm is quantifying Na[^18^F]F PET in SpA patients and knowing that VOI definition depends on the specific clinical question at hand, a strategy was developed for the joint segmentation. In particular, the FJs, CVJs and CTJs were segmented not as volumes but as single points representing the joint center. To establish an appropriate VOI, a dilation operation was applied to this central point using a spherical structuring element with a radius of 10mm [33, 34].

### Morphological method

**Fig. 1:**
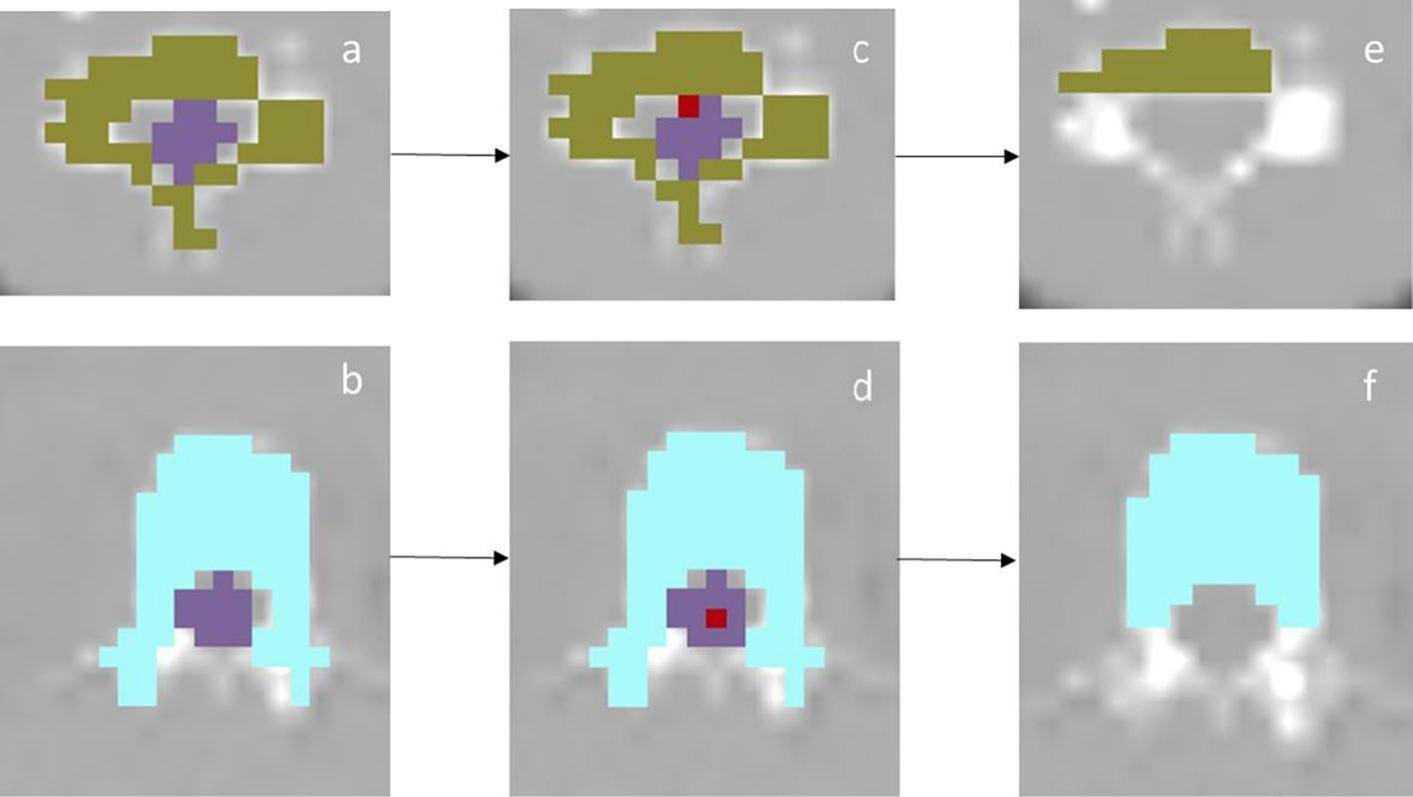
Morphological segmentation of the vertebral bodies (VBs). **a**, **b** Axial slice with initial segmentation of C4 (Green) (**a**) and L1 (Blue) (**b**) with the spinal cord (Purple). **c** Most anterior point of the spinal cord (Red). **d** Central point of spinal cord (Red). **e**, **f** VB segmentation, only containing pixels anterior of the spinal cord point

For the first method, the segmented vertebrae were separated into anterior and two posterior parts using Matlab R2017b. The first step was to separate the vertebral bodies (VBs) from the transverse and spinal processes. For each axial slice, the spinal cord was localized and all vertebrae voxels anterior to this structure were classified as VB, whereas the remaining voxels were classified as the posterior vertebra. In the case of the cervical vertebrae and the three upper thoracic vertebrae, the most anterior pixel of the spinal cord was chosen because of the relatively anterior position of the transverse processes in these vertebrae (Fig. 1a, c, e). For the lower nine thoracic vertebrae and the lumbar spine, the center of the spinal cord was localised, and all voxels anterior to this location were segmented as VBs (Fig. 1b, d, f).

The remaining posterior vertebrae were further divided into left and right parts, using the center of mass (COM) of the spinal cord segmentation. Voxels to the left of the sagittal plane of the COM were classified as the left posterior vertebra (LPV), while voxels to the right were classified as the right posterior vertebra (RPV). The IVDs and posterior joints were segmented using these three bone segmentations with a variation of the method developed by Piri et al*.* [24].

To acquire the IVD segmentations, the two adjacent VBs were dilated using a vertical structuring element (length 12 mm for lumbar, 8 mm for thoracic, and 4 mm for cervical spine). After dilation, the overlapping voxels of the VBs that were not part of the initial bone segmentation were classified as IVD. A similar dilation procedure was iteratively applied to the LPV and RPV for the FJ segmentation, as well as to the ribs for the CVJs and CTJs and the sacrum and ilium for the SIJs. A 4 mm radius spherical structuring element, which corresponded to the voxel size, was used. The center of the joint was determined by calculating the COM of the overlapping dilated voxels.

### Atlas-based method

**Fig. 2:**
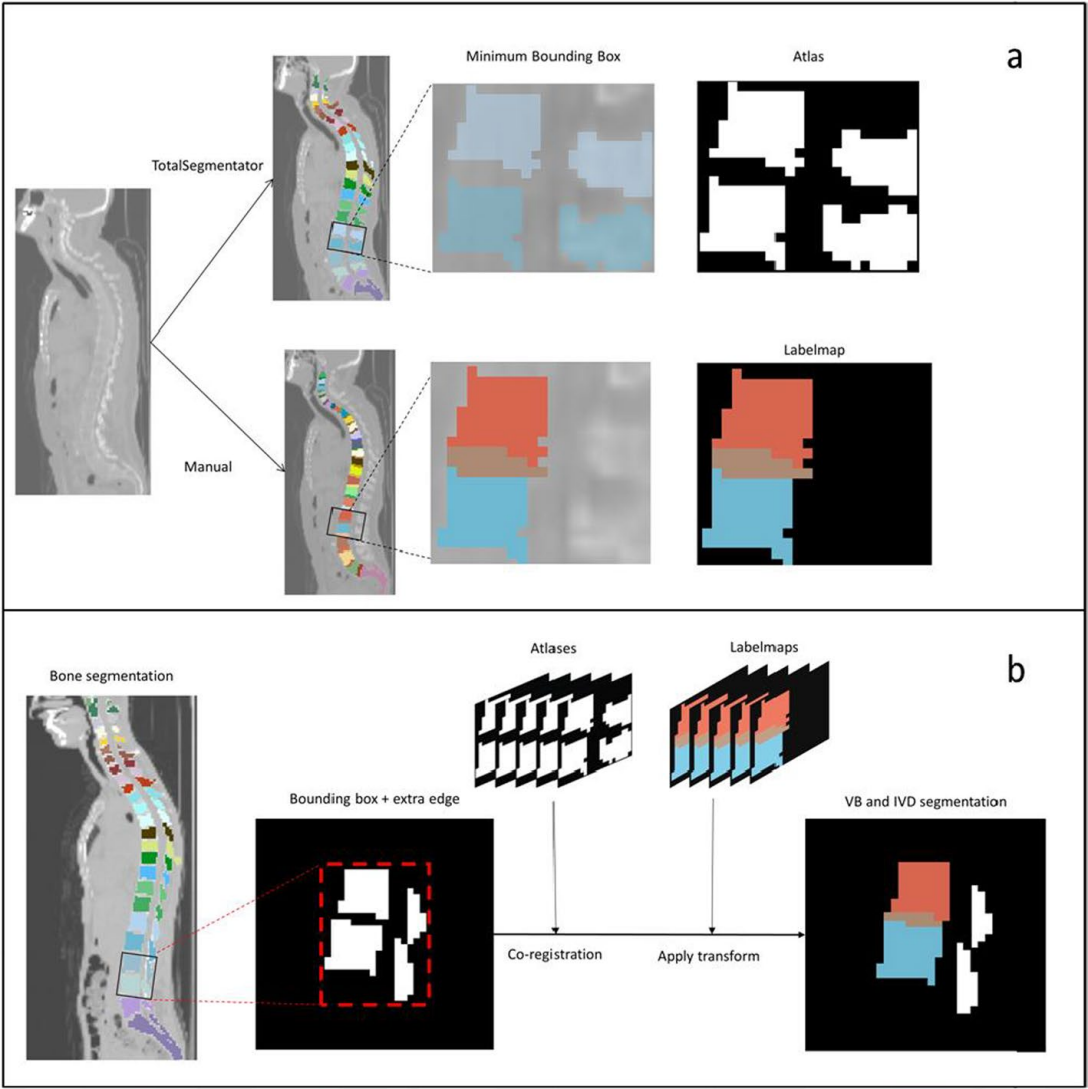
Atlas-based method. **a** Atlas creation. **b** Atlas-based segmentation. VB: Vertebral body, IVD: Intervertebral disk

The atlas-based method involved manual segmentation of five LDCT scans for use as atlases. Five atlases were considered sufficient to account for anatomical variations, while not overly complicating the co-registration process [35]. To ensure diversity in atlas selection, the distance between the COMs of C1 and the sacrum in the bone segmentations was calculated for all available LDCT scans, providing an estimate of the spinal length. The LDCT scans of the patients that represented the 10th, 30th, 50th, 70th, and 90th percentiles of the spinal length were used as atlases. These images were further processed using 3D Slicer v5.0.2 [36] and Matlab R2017b. To optimise co-registration of all structures, the atlases were divided into bounding boxes containing sets of two adjacent vertebrae, or the sacrum and the left or right ilium in the case of SIJ segmentation. The same bounding boxes were applied to the manually segmented label maps, resulting in 26 partial atlases and label maps (Fig. 2a).

To segment the structures on a target LDCT scan, the adjacent vertebrae bounding boxes were obtained using TotalSegmentator v2. A 10-voxel zero padding layer was added around the cropped image to ensure optimal co-registration. This prevented uncontrolled scaling of the moving image outside the fixed image. For each vertebral level, the five corresponding atlases were co-registered onto the target images using the Elastix toolbox (v4.8) [37] (Fig. 2b). A similarity transform was applied to the atlases, optimized through 100 iterations using stochastic gradient descent as the optimizer and advanced mean squares as the metric. The final transform of each atlas was applied to the corresponding label map. Majority voting was employed for the final VB, IVD, and SIJ segmentation. For the localization of the posterior joints, the mean coordinates of the five estimated locations were calculated. The bounding boxes containing the final segmentations were re-integrated into the target image.

### DVU composition

**Fig. 3:**
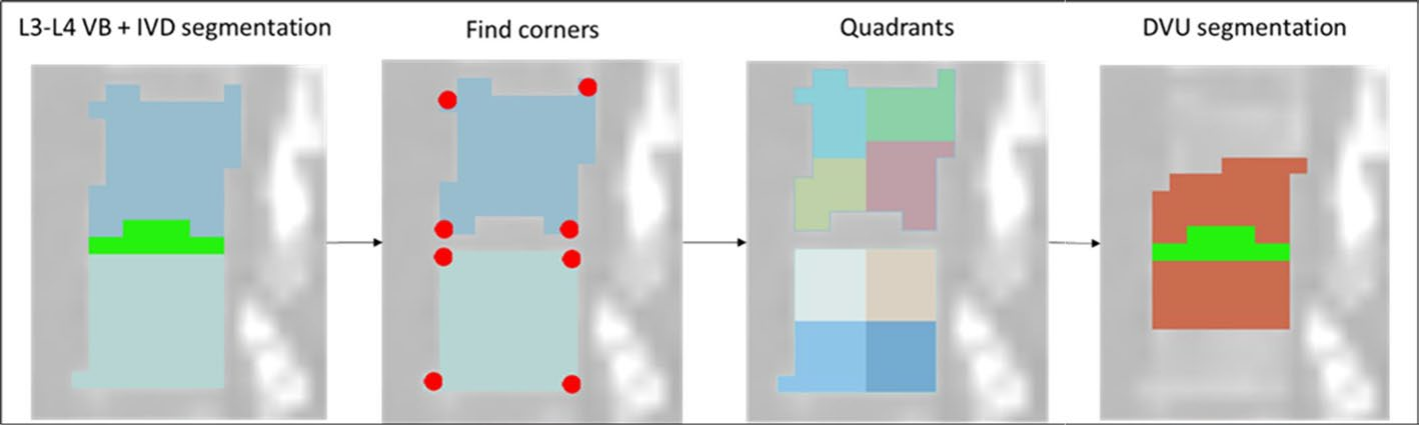
Composition of the disco vertebral unit segmentation. VB: Vertebral Body, DVU: Discovertebral unit

The final step to obtain the DVU segmentation was to combine the IVD, the lower half of the upper VB and the upper half of the lower VB into one segmentation. For each vertebral body, the COM was calculated. The sagittal plane of the COM was used to determine the corners of the VB. Then, all pixels of the 2D image were assigned to the closest corner. Different labels were assigned to the VB left and right of the central sagittal plane, thus dividing each VB into 8 quadrants. The four lower quadrants of each vertebra were combined with the IVD and the four upper quadrants of the lower vertebrae (Fig. 3).

### Validation

The validation of the segmentation methods involved evaluating the segmentation performance, reproducibility, and lesion detection accuracy. Ten randomly chosen LDCT scans from the SpA cohort not being used as an atlas were segmented using both methods to assess the performance. The manual segmentation for ground truth definition was performed by a clinical researcher (WvdH), supervised by an experienced board-certified nuclear medicine specialist (GZ). The evaluation metrics included the average Hausdorff distance (HD) and Dice similarity coefficient (DSC) for the DVUs and SIJs, as well as the distance between the estimated point and the manually segmented point for the FJs, CVJs and CTJs. The HD and DSC are both metrics that indicate the overlap between the estimated segmentation and the ground truth. While the DSC is widely used and provides a clear measure of voxel-wise accuracy, it does not account for the distance between misclassified voxels and the ground truth. In contrast, the HD captures the distance between the incorrectly segmented voxels and the ground truth, making it particularly suitable for this application, given the small volume of the segmented structures. For consistency with other research, we report the DSC alongside the HD. Additionally, for posterior joints, where only the center of the joint is segmented as a single voxel, overlap metrics like DSC and HD are not applicable. Instead, the error distance is used to quantify the segmentation accuracy in these cases.

### Reproducibility

To assess reproducibility, the segmentation methods were applied on 16 test–retest LDCT scans of eight lung cancer patients with no spinal metastases [38]. These patients underwent [^18^F]FDG PET/LDCT scans twice within a 3-day time interval. The spine segmentations from the second scan were co-registered onto the spine from the first scan using a rigid transformation. The same transformation was applied to the label map, and the similarity between the results was assessed using DSC, average HD, and mean error distance.

### Statistical analysis

The median and inter-quartile range (IQR) of the performance metrics were calculated for all anatomical locations in both methods. Since no normality was assumed, and to be robust for outliers, the results were compared using a Wilcoxon rank sum test. Differences in performance were considered significant when the p-value was lower than 0.05. To provide additional context on the magnitude of the differences, effect sizes (ES) were calculated by finding the differences in performance between the two methods for each individual anatomical structure. The median difference and IQR were reported alongside the *p* value. The metrics were calculated for all DVUs and all posterior joints combined, as well as for each group separately, to determine the differences in performance between the two segmentation methods.

### Lesion detection

To identify the optimal metrics and thresholds for automated lesion detection, a nuclear medicine specialist (GZ) and a musculoskeletal radiologist (either RH or TD) performed visual assessment of each anatomical structure, classifying them as either positive or negative for pathological tracer uptake. Importantly, this method was not intended for PET lesion segmentation but was instead used for detection of pathological uptake presence. Cases of differences between the readers were solved by an independent reader (CvdL). Both segmentation methods were applied to the LDCTs, and for each VOI, the maximum, peak and mean SUVs (SUVmax, SUVpeak and SUVmean) were extracted from the PET images. Next, the area’s under the receiver operator curves (AUC) were assessed for lesion detection performance analysis, compared with visual assessment. To correct for background uptake, the target-to-background ratio (TBR) of the best-performing segmentation method and SUV metric corrected by the median SUV (SUVmedian) of the spine, spinal segments, femur, liver or aortic blood pool were assessed. Since background uptake in the spine differs among the regions, the SUVmedian of the cervical, thoracic and lumbar spine were individually extracted. These corrections were applied to the SUV metrics that were extracted from the VOIs, depending on the VOIs location, leading to three different background correction factors for each scan. The background uptakes were extracted using the initial TotalSegmentator segmentations, applying one-voxel erosion procedures to the femur and aorta to remove cortical bone and vascular lumen, respectively, and accounting for misalignment between PET and CT. The threshold yielding the highest accuracy in the best-performing method was calculated using the Kolmogorov–Smirnov (K-S) statistic. The differences between the AUC curves were assessed using the DeLong test.

Using the optimal thresholds found in the AUC analysis, the baseline scans were evaluated to determine the sensitivity and specificity of the automated method at a lesion-level. Additionally, discrepancies between the visual assessment and the automated method were analysed to identify potential causes and areas for improvement.

## Results

Forty-five baseline (prior to initiation or switch of treatment) scans of SpA patients who underwent Na[^18^F]F PET/CT, were analysed. All images underwent segmentation using the TotalSegmentator algorithm. The mean vertical distance between the COM of the sacrum and the COM of the upper vertebra was 58.1 (± 3.8) cm. All lengths were sorted from small to large, and those at the 5th, 14th, 23rd, 32nd, and 39th indexes were selected as the atlases.

**Fig. 4:**
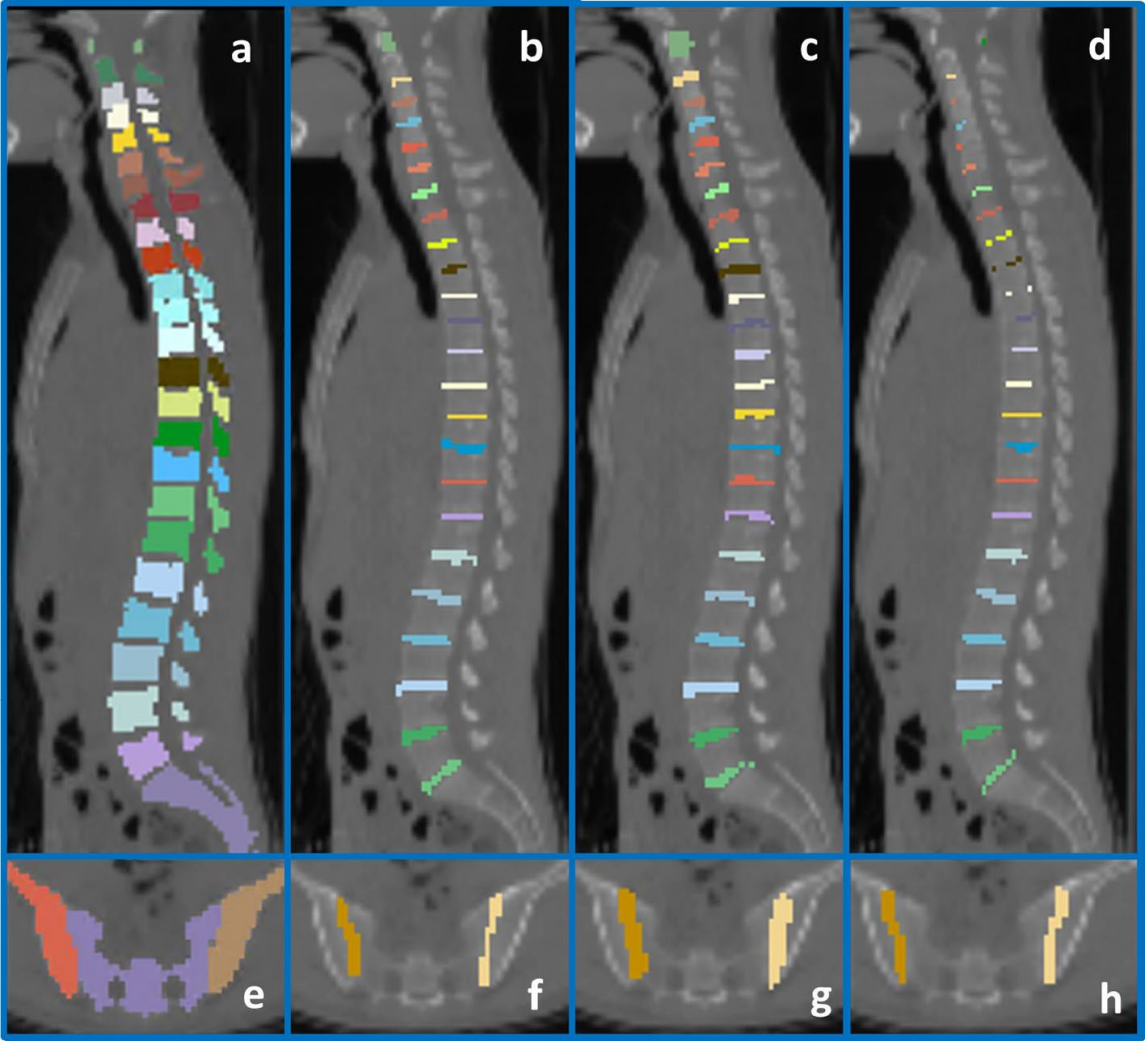
Segmentation results for the intervertebral disks (**a**–**d**) and the sacro-iliac joints (**e**–**h**). **a**, **e** Initial segmentation. **b**, **f** Manual segmentation. **c**, **f** Atlas-based segmentation. **d**, **h** Morphological segmentation

**Fig. 5:**
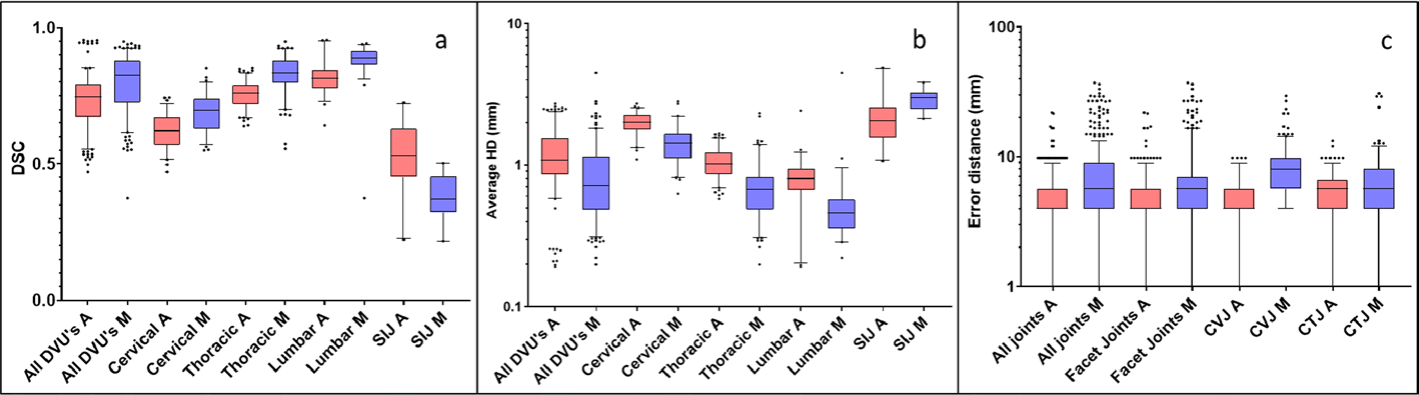
Boxplots of the segmentation performance metrics for the atlas-based method (Red) (A) and the morphological method (Blue) (M), compared to the manual segmentation. **a** Dice similarity coefficients (DSC). **b** Average Hausdorff distance (HD). **c** Error distance between joint centers. DVU: Discovertebral Unit, SIJ: Sacro-iliac joint, CVJ: Costovertebral joint, CTJ: Costotransverse joint

The results of the IVD and SIJ segmentation are visualized in Fig. 4. In the atlas-based method, the estimated IVD volumes were larger than the manual segmentation in most cases (Fig. 4c). In contrast, the morphological method (Fig. 4d) led to visually smaller volumes compared to the manual segmentation (Fig. 4b). The segmentation performance for the DVUs and SIJs is presented in Fig. 5a and b. For the segmentation of all DVUs, the morphological method performed significantly better than the atlas-based method, with a DSC of 0.82 (IQR: 073–0.88) compared to 0.74 ((0.68–0.79); *p* < 0.001, ES = 0.07 (0.02–0.11)). The atlas-based method achieved a considerably higher average HD of 1.08 (0.85–1.55) for all DVUs, compared to 0.74 (0.68–0.79) for the morphological method (*p* < 0.001, ES = 0.38 (0.10–0.58)). For the SIJ segmentation, the atlas-based method reached a better average DSC (0.53 (0.45–0.63) vs. 0.37 (0.32–0.45); *p* = 0.001, ES = 0.18 (0.02–0.26)) and HD (2.04 (1.57–2.56) vs. 3.01 (2.49–3–25); *p* = 0.001, ES = 0.98 (0.11–1.49)) (Supplement 1). For the posterior joints, the atlas-based method achieved a significantly lower distance between the manual segmented joint center, compared to the morphological method for all joints combined (4.00 (4.00–5.66) vs. 5.66 (4.00–8.00); *p* < 0.001, ES = 0.00 (− 1.27–4.00)), and for each set of joints individually (FJs and CVJs: *p* < 0.001, ES = 0.00 (− 1.27–3.29) and 4.00 (1.66–5.67), respectively, CTJs: *p* = 0.014, ES = 0.00 (− 1.66–2.96)) (Fig. 5c, Supplement 2). Values of 4.00 and 5.66 are frequently represented in the error distance tables. These values represent segmentations that estimate the joint center one voxel next to, or diagonally next to the manual segmentations. As can be deduced from the IQRs, at least 75% of all joint center segmentations fell within this range for the atlas-based method.

### Reproducibility

The reproducibility metrics are presented in the supplementary files. The atlas-based method achieved significantly better performance for most structures and metrics. The median average HD for all DVUs combined was 0.80 (0.48–1.08) for the atlas-based method compared to 0.85 (0.55–1.15) for the morphological method (*p* < 0.001, ES = 0.09 (− 0.06–0.36)) (Supplement 3, 4). For the combination of the FJs, CVJs and CTJs the atlas-based method yielded a median error distance of 4.00 (0.00–5.66). For the morphological method the reproducibility of the joint segmentation was significantly worse for all joints (*p* < 0.001, ES = 1.27 (0.00–4.00)) and all individual groups of joints (FJs, CVJs and CTJs: *p* < 0.001, ES = 1.27 (− 0.21–4.00), 1.66 (0.00–4.34), 0.00 (0.00–4.00), respectively) (Supplement 5).

### Lesion detection

**Fig. 6:**
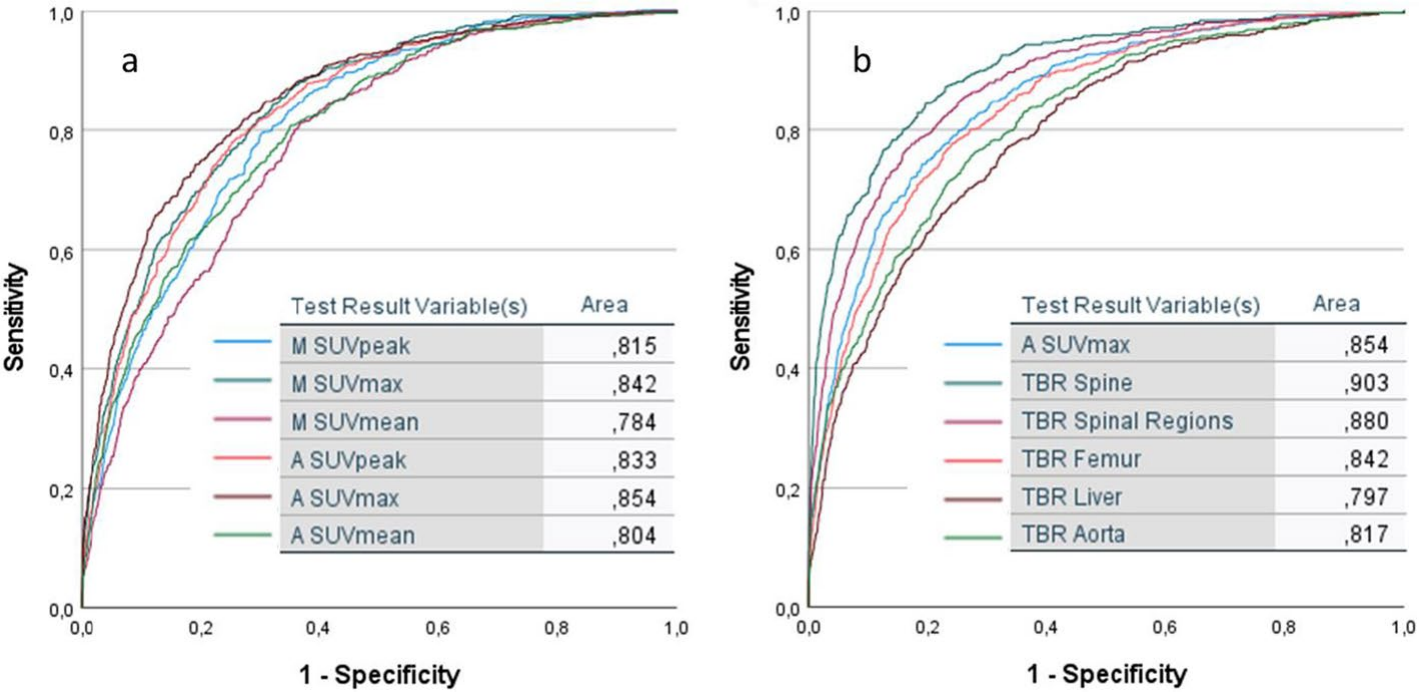
Receiver operator curves of the different metrics applied for optimal lesion detection performance. **a** Comparison of SUVmax, SUVmean, and SUVpeak for both the atlas-based method (A) and the morphological method (M). **b** Comparison of the best-performing metric SUVmax in the atlas-based segmentation corrected by the SUVmedian of different possible background structures

A total of 53 Na[^18^F]F PET/CT scans were analysed, distributed over 17 patients who underwent three scans, and one patient who did not undergo the final follow-up scan at 48 weeks. This led to 6,254 VOIs, of which 567 (9.1%) were classified as Na[^18^F]F PET positive by the readers. The VOIs that were generated by the atlas-based method performed consistently better than the VOIs that were generated by the morphological method (atlas-based: AUC = 0.85, 0.83, 0.80 for SUVmax, SUVpeak and SUVmean respectively, vs. AUC = 0.84, 0.82 and 0.78 for the morphological method) (Fig. 6a). Furthermore, the atlas-based classification method that used the SUVmax performed significantly better than the other metrics SUVpeak and SUVmean (AUC = 0.85 vs. 0.83 and 0.80; both *p* < 0.001) (Fig. 6a). When the TBR of the spine and the spinal segments individually were used, the classification performance improved significantly (AUC = 0.90 and 0.88 vs 0.85, respectively; both *p* < 0.001) (Fig. 6b). Correction for background uptake in the femur, liver, and aorta decreased detection accuracy to AUCs of 0.84, 0.80, and 0.82, respectively. Separate analysis of the joints and the DVUs using the TBR yielded AUCs of 0.93 for the posterior joints and an AUC of 0.89 for the DVUs. The optimal K-S statistic was found at a cut-off value of 1.8 times the spine TBR for the posterior joints and SIJs, and 2.25 for the DVUs. When the uncorrected SUVmax was used, the optimal cut-offs for detecting positive VOIs were respectively 10.4 and 13.0.

### Evaluation of diagnostic performance

In the 18 baseline scans, a total of 2,124 anatomical locations were evaluated, with 223 locations identified as showing pathological uptake based on visual assessment. The atlas-based method achieved a lesion detection sensitivity of 86.1% and specificity of 89.0%, while the morphological method showed a sensitivity of 83.4% and specificity of 87.7% (Supplement 6,7). Upon reviewing all discrepancies between the automated methods and the visual assessment, the most common error occurred in cases of visually correct segmentations with elevated uptake that did not meet the threshold to be classified as pathological by the visual readers (71.0% and 62.7% for the atlas-based and morphological methods, respectively). The second most frequent source of discrepancies involved segmentation errors, which led to either overestimation or underestimation of tracer uptake within a structure (14.9% for the atlas-based method and 24.4% for the morphological method). Another notable cause of error was the over-projection of tracer uptake into nearby segmentations, particularly in those cases where pathological uptake in CVJs projected into DVU or FJ segmentations. These accounted for 14.1% of errors in the atlas-based method and 12.9% in the morphological method. Most discrepancies were found in the DVUs and CVJs, with a notably higher number of discrepancies caused by errors in the morphological segmentations of the CVJs (n = 37) compared to the errors in the atlas-based method (n = 9) (Supplement 8).

## Discussion

This paper describes the first fully automated methods for detailed segmentation of the axial skeleton on LDCT images. These segmentations were employed for automated detection of Na[^18^F]F PET-positive lesions. The atlas-based method showed better reproducibility (error distance of posterior vertebral joints of 4.00 vs. 5.66 mm) and lesion detection (AUC of 0.85 vs 0.84), and is thus chosen to be the best method.

In the DVU and SIJ segmentation, the atlas-based method segmented visually larger volumes than the manual segmentation, whereas the morphological method yielded visually smaller DVU and SIJ volumes. Even though the morphological methods showed better DSCs (0.82 vs. 0.74 for the atlas-based method), the lesion detection performance was better using the atlas-based method. This might be explained by the over-segmentation of structures in the atlas-based method, which increased detection sensitivity and reduced the possible influence of segmentation errors or misalignment between the PET and LDCT. A limitation of using DSC and HD as segmentation metrics is that they do not differentiate between over-segmentation and under-segmentation compared to the ground truth. This can lead to situations where a method with higher segmentation accuracy underperforms in terms of clinical utility. In this study, while the morphological method demonstrated better alignment with the ground truth, the atlas-based method achieved a higher AUC in lesion detection. This indicates that, for the specific application of detecting SpA lesions on Na[^18^F]F PET, the AUC is a more appropriate metric for evaluating segmentation performance, as it directly reflects the method's ability to identify clinically relevant tracer uptake. This method can facilitate more reproducibility in lesion detection but requires visual assessment (a human-in-the-loop approach) to verify the detection results.

In both methods, the segmentation performances of the small structures, such as the cervical DVUs and the sacro-iliac joints, were considerably lower than the larger structures (DSC of 0.62 for cervical DVUs and 0.53 for the SIJs, compared to 0.81 in lumbar DVUs for the atlas-based method). In the segmentation of the cervical vertebrae, which have a volume of around 10 cm^3^ [39], an error of one wrongly segmented voxel had a larger impact on the total segmentation performance compared to the thoracic and lumbar vertebrae, which have a volume of ± 20 cm^3^ and ± 40 cm^3^ [40], respectively. The low performance of SIJ segmentation, especially compared to the previously reported performances of the deep learning based methods developed by Zhang and Van den Berghe (DSCs of 0.89 and 0.75 respectively, vs 0.53 in our study), can also be explained by the intrinsic properties of the evaluation metrics, that are susceptible to minor errors in thin structures [25, 26]. The resolution in which the SIJs were segmented in our study was compatible with the PET resolution of 4 × 4 × 4 mm^3^, whereas the aforementioned studies utilized high-resolution CT to achieve detailed surface segmentation for detecting erosions and ankyloses, essential for grading and diagnosing sacroiliitis. For such applications, a direct UNet based approach may be more suitable, as it provides greater detail and precision compared to the indirect, less detailed methods used in our study. The complex anatomy and inter-patient variability of the SIJs further challenge the suitability of the atlas-based method. AI-based segmentation approaches, such as deep learning models, are better equipped to adapt to these anatomical variations. Also, non-rigid co-registration in the atlas-based method might improve segmentation performance. However, for PET lesion detection, the over-segmentation by the atlas-based method may be beneficial in achieving better sensitivity in the lesion detection task. This highlights the trade-off between segmentation accuracy and application-specific performance, depending on the context.

Visual assessment as a gold standard has limitations due to its subjectivity and variability between readers, leading to inconsistencies in identifying subtle or borderline lesions. It is also time-consuming and prone to fatigue-related errors, particularly when analysing large datasets. In this study the visual assessment may have led to under-estimation of the automated lesion detection performance, by possibly missing lesions that were recognised by the automated methods. These limitations highlight the need for automated approaches to provide consistent, objective, and scalable assessments.

Another limitation of this study is the choice of spherical volumes used as posterior joint segmentations. Due to the low resolution and ankyloses in the SpA LDCTs, no clear joint surfaces were visible in some cases. A fixed-size spherical volume was placed on the estimated center of the joint to obtain an adequate segmentation. Based on the limited literature about the proportions of posterior joint VOIs [33, 34] a spherical VOI with a diameter of 20 mm was chosen. This is slightly larger than the described dimensions for the lumbar and cervical FJs, but it was chosen because adequate sensitivity is important in this application. Additionally, the lack of visible IVD surfaces in some cases made manual segmentation less reliable as a comparison. Advances in PET imaging technology are expected to enable imaging with higher sensitivity, allowing for more precise and detailed segmentation of structures of interest. Additionally, the implementation of advanced techniques to correct for misalignment between PET and CT has the potential to further enhance lesion detection performance.

The segmentation methods were explicitly developed for detecting Na[^18^F]F PET-positive lesions in SpA patients, assisting nuclear medicine physicians by highlighting regions of possible pathological uptake, potentially improving sensitivity, reproducibility, and reducing assessment time. Also, the segmentations can be used for other applications requiring a detailed axial skeleton segmentation. With the emergence of LDCT as a stand-alone modality in SpA diagnosis, automated segmentation may aid in the quantification and detection of SpA [41, 42]. Beyond SpA, the segmentation methods can be used in other diseases and PET tracers, such as [^18^F]FDG, to analyse and classify spinal malignancies.

Further steps are necessary to achieve fully automated PET quantification, including direct segmentation of Na[^18^F]F PET-positive lesions, for which the lesion detection algorithm described in this study may be a useful first step. A deep learning based method seems the most appropriate approach for Na[^18^F]F PET lesion segmentation. The features of these lesions can potentially be used for differentiation between SpA and degenerative disease or to predict treatment response through machine learning approaches. This would assist rheumatologists in earlier and more accurate diagnosis of SpA, and facilitate more personalized treatment decisions based on PET imaging features that may stratify patients into groups.

## Conclusion

In summary, we present the first automatic methods for segmentation of all spinal DVUs and joints on LDCT, which can be used for accurate detection of Na[^18^F]F PET-positive lesions in SpA patients. An atlas-based approach, combined with a threshold-based detection algorithm using the SUVmax corrected by the SUVmedian of the spine emerges as the most promising approach, exhibiting the best reproducibility and lesion detection performance. More research is warranted on the segmentation of the positive lesions, and its implications for the diagnosis and treatment strategy in SpA and beyond.

## Supplementary Information

Additional file 1.

## Data Availability

The datasets used and/or analysed during the current study are available from the corresponding author on reasonable request.

## Abbreviations

AUC: Area Under the Curve
COM: Center of Mass
CT: Computed Tomography
CTJ: Costotransverse Joints
CVJ: Costovertebral Joints
DSC: Dice Similarity Coefficient
DVU: Discovertebral Unit
ES: Effect Size
FDG: Fluorodeoxyglucose
FJ: Facet Joints
HD: Hausdorff Distance
IQR: Inter-Quartile Range
IVD: Intervertebral Disk
K-S: Kolmogorov–Smirnov
LDCT: Low-Dose Computed Tomography
LPV: Left Posterior Vertebra
MBq: MegaBecquerel
NaF: Sodium Fluoride
PET: Positron Emission Tomography
RPV: Right Posterior Vertebra
SIJ: Sacro-Iliac Joint
SpA: Spondyloarthritis
SUV: Standardized uptake value
TBR: Target to Background Ratio
VB: Vertebral Body
VOI: Volume of Interest
