# Supplementary material for: Automated segmentation of the sacro-iliac joints, posterior spinal joints and discovertebral units on low-dose computed tomography for Na[18F]F PET lesion detection in spondyloarthritis patients
Source: EJNMMI Phys. 2025 Mar 10;12:20. doi: 10.1186/s40658-025-00734-7 (PMC11891110; doi:10.1186/s40658-025-00734-7)
Supplement: Supplementary file 1 — Additional file 1. [file 40658_2025_734_MOESM1_ESM.docx]

Supplementary material

|  | Median average HD (IQR) (mm) | | |  | Median DSC (IQR) | | | |
| --- | --- | --- | --- | --- | --- | --- | --- | --- |
|  | Atlas | Morphological | p | Effect Size | Atlas | Morphological | p | Effect Size |
| *All DVUs* | 1.08 (0.85-1.55) | 0.71 (0.49-1.15) | <0.001 | 0.38 (0.10-0.58) | 0.74 (0.68-0.79) | 0.82 (0.73-0.88) | <0.001 | 0.07 (0.02-0.11) |
| *Cervical* | 2.00 (1.78-2.26) | 1.42 (1.12-1.67) | <0.001 | 0.61 (0.27-0.88) | 0.62 (0.57-0.67) | 0.70 (0.63-0.74) | <0.001 | 0.09 (0.03-0.13) |
| *Thoracic* | 1.02 (0.86-1.22) | 0.67 (0.49-0.82) | <0.001 | 0.39 (0.18-0.52) | 0.76 (0.72-0.79) | 0.83 (0.80-0.88) | <0.001 | 0.08 (0.05-0.12) |
| *Lumbar* | 0.80 (0.67-0.94) | 0.46 (0.36-0.56) | <0.001 | 0.31 (0.06-0.43) | 0.81 (0.78-0.85) | 0.89 (0.87-0.91) | <0.001 | 0.07 (0.00-0.09) |
| *SIJ* | 2.04 (1.57-2.56) | 3.01 (2.49-3.25) | 0.001 | 0.98 (0.11-1.49) | 0.53 (0.45-0.63) | 0.37 (0.32-0.45) | 0.001 | 0.18 (0.02-0.26) |

***Sup. 1*** *Performance metrics for discovertebral unit and sacro-iliac joint segmentation quality compared to manually segmented ground truth. HD: Hausdorff distance, SIJ: Sacro-iliac joint.*

|  | Median error distance (IQR) (mm) | | | |
| --- | --- | --- | --- | --- |
|  | Atlas | Morphological | p | Effect Size |
| All | 4.00 (4.00-5.66) | 5.66 (4.00-8.00) | <0.001 | 0.00 (-1.27-4.00) |
| FJs | 4.00 (4.00-5.66) | 5.66 (4.00-6.93) | <0.001 | 0.00 (-1.27-3.29) |
| CVJs | 4.00 (4.00-5.66) | 8.00 (5.66-9.80) | <0.001 | 4.00 (1.66-5.67) |
| CTJs | 5.66 (4.00-6.61) | 5.66 (4.00-8.00) | 0.014 | 0.00 (-1.66-2.96) |

***Sup. 2*** *Mean error distance between the location estimated by both automatic methods and the manually localised ground truth. FJs: Facet joints, CVJs: Costovertebral joints, CTJs Costotransverse joints.*

*
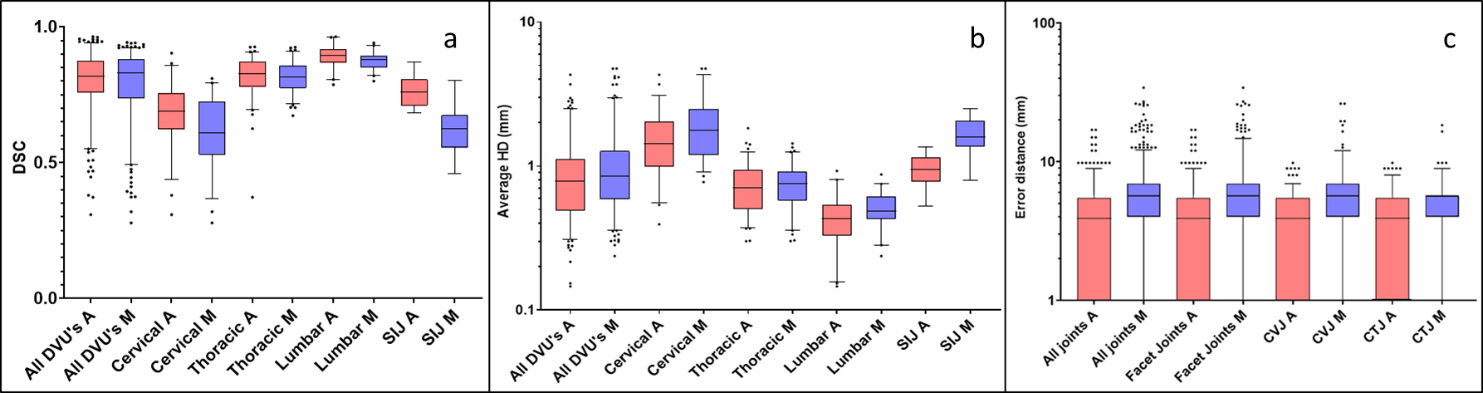
*

***Sup. 3*** *Boxplots of the segmentation reproducibility for the atlas-based method (Red) (A) and the morphological method (Blue) (M) in the test-retest assessment. a: Dice similarity coefficients (DSC). b: Average Hausdorff distance (HD). c: Error distance between joint centers. DVU: Discovertebral Unit, SIJ: Sacro-iliac joint, CVJ: Costovertebral joint, CTJ: Costotransverse joint*

|  | Median average HD (IQR) (mm) | | |  | Median DSC (IQR) | | | |
| --- | --- | --- | --- | --- | --- | --- | --- | --- |
|  | Atlas | Morphological | P | Effect Size | Atlas | Morphological | p | Effect Size |
| *All DVUs* | 0.80 (0.48-1.08) | 0.85 (0.55-1.15) | <0.001 | 0.09 (-0.06-0.36) | 0.81 (0.74-0.88) | 0.80 (0.73-0.87) | 0.13 | 0.01 (-0.02-0.05) |
| *Cervical* | 1.31 (0.97-1.74) | 1.65 (1.17-2.02) | <0.001 | 0.33 (0.05-0.72) | 0.69 (0.62-0.76) | 0.64 (0.57-0.73) | <0.001 | 0.06 (-0.01-0.13) |
| *Thoracic* | 0.71 (0.50-0.94) | 0.75 (0.58-0.91) | 0.23 | 0.04 (-0.09-0.19) | 0.83 (0.78-0.87) | 0.81 (0.77-0.86) | 0.07 | 0.01 (-0.02-0.03) |
| *Lumbar* | 0.43 (0.33-0.54) | 0.49 (0.43-0.62) | 0.04 | 0.02 (-0.05-0.14) | 0.89 (0.87-0.92) | 0.88 (0.85-0.89) | 0.04 | 0.01 (-0.01-0.03) |
| *SIJ* | 0.97 (0.81-1.19) | 1.60 (1.34-2.04) | <0.001 | 0.75 (0.58-0.95) | 0.76 (0.71-0.81) | 0.62 (0.55-0.67) | <0.001 | 0.14 (0.11-0.19) |

***Sup. 4*** *Performance metrics for discovertebral unit (DVU) and sacro-iliac joint (SIJ) segmentation quality on test-retest LDCT scans that were acquired with a time interval of three days. HD: Hausdorff distance.*

|  | Median error distance (IQR) (mm) | | | |
| --- | --- | --- | --- | --- |
|  | Atlas | Morphological | P | Effect size |
| All | 4.00 (0.00-5.66) | 5.66 (4.00-6.93) | <0.001 | 1.27 (0.00-4.00) |
| FJs | 4.00 (0.00-5.66) | 5.66 (4.00-6.93) | <0.001 | 1.27 (-0.21-4.00) |
| CVJs | 4.00 (0.00-5.66) | 5.66 (4.00-6.93) | <0.001 | 1.66 (0.00-4.34) |
| CTJs | 4.00 (0.00-5.66) | 5.66 (4.00-5.66) | <0.001 | 0.00 (0.00-4.00) |

***Sup. 5*** *Mean error distance between the location estimated by both automatic methods on the test and retest LDCT. FJs: Facet joints, CVJs: Costovertebral joints, CTJs Costotransverse joints.*

| Atlas | Positive in automated method | Negative in automated method | Total |
| --- | --- | --- | --- |
| Positive in visual assessment | 192 (9.0%) | 31 (1.5%) | 223 (10.5%) |
| Negative in visual assessment | 210 (9.9%) | 1691 (79.6%) | 1901 (89.5%) |
| Total | 402 (18.9%) | 1722 (81.1%) | 2124 |

***Sup. 6*** *Confusion Matrix of the lesions detected by the atlas-based method compared with visual assessment.*

| Morphological | Positive in automated method | Negative in automated method | Total |
| --- | --- | --- | --- |
| Positive in visual assessment | 186 (8.8%) | 37 (1.7%) | 223 (10.5%) |
| Negative in visual assessment | 234 (11.0%) | 1667 (78.4%) | 1901 (89.5%) |
| Total | 420 (19.8%) | 1704 (80.2%) | 2124 |

***Sup. 7*** *Confusion Matrix of the lesions detected by the morphological method compared with visual assessment.*

|  | Atlas | | | Morphological | | |
| --- | --- | --- | --- | --- | --- | --- |
|  | Threshold | Segmentation | Over-Projection | Threshold | Segmentation | Over-Projection |
| *DVUs* | 60 | 10 | 24 | 59 | 10 | 26 |
| *FJs* | 23 | 11 | 9 | 23 | 9 | 6 |
| *CVJs* | 71 | 9 | 1 | 72 | 37 | 3 |
| *CTJs* | 12 | 6 | 0 | 12 | 6 | 0 |
| *SIJs* | 5 | 0 | 0 | 4 | 4 | 0 |
| *Total* | 171 | 36 | 34 | 170 | 66 | 35 |

***Sup. 8*** *Overview of the locations and causes of discrepancies between the visual assessment and the atlas-based and morphological automated methods. Threshold: The segmentation is correct and there is increased tracer uptake, but the location is not marked as pathological in the visual assessment. Segmentation: An error in CT-based automated segmentation has caused incorrect PET quantification. Over-Projection: Segmentation is correct but tracer uptake in a nearby location projects into volume of interest.*
